# Examining Second Language Listening, Vocabulary, and Executive Functioning

**DOI:** 10.3389/fpsyg.2020.01122

**Published:** 2020-06-03

**Authors:** Matthew P. Wallace, Kerry Lee

**Affiliations:** ^1^Department of English, University of Macau, Macau, China; ^2^Department of Early Childhood Education, The Education University of Hong Kong, Tai Po, Hong Kong

**Keywords:** second language listening, executive functioning (EF), second language vocabulary, updating, shifting

## Abstract

Performance on second language (L2) listening tests is influenced by individual differences in listener characteristics (e.g., executive functioning and vocabulary size) and characteristics of the listening measure (e.g., text length or skills measured). For listeners, the amount of linguistic knowledge is most important for comprehension outcomes. As language proficiency increases, non-linguistic factors, like the executive functions (EF) of working memory, purportedly begin to exert influence on listening performance. EF represents the range of functions performed by the central executive (the processing component) of the working memory system and have largely been studied in the context of updating (revising information held in temporary storage) and shifting (switching attentional focus among mental representations). To test these theoretical claims, the relationship among L2 listening, vocabulary size, updating, and shifting was examined. This included a moderation analysis to investigate whether the relationship between EF and listening was dependent upon vocabulary size. The relationships among the variables were also examined for varied test characteristics to see if contributions from EF and vocabulary differed according to text length or skill measured. In total, 209 Japanese senior high school EFL learners completed a standardized listening test and tests measuring updating, shifting, and vocabulary size. Results from structural equation modeling showed that only vocabulary was predictive of listening performance, regardless of text length or skill measured on the test. Results also showed that vocabulary size did not moderate the relationship between EF and listening, suggesting that the non-linguistic factors were not important for listening regardless of vocabulary size. The findings support claims that linguistic knowledge is most important for listening and that non-linguistic factors are less important for low-level listeners. The findings also contribute empirical evidence for the relationship between L2 listening and EF, a novel conceptualization of the working memory construct.

## Introduction

Comprehension of second language (L2) speech is a complex cognitive process that involves mental processing and the use of knowledge resources to interpret what is said. Listening tests measuring comprehension are designed to gauge how efficiently test takers utilize these cognitive resources to accomplish listening tasks, like identifying specific information from speech. Performance on listening tests may therefore be attributed to individual differences in characteristics of the listener (e.g., vocabulary size and working memory) or those of the listening task (e.g., response format) (Buck, 2001). Research investigating listening assessment has mainly focused on how listener characteristics influence performance (e.g., Andringa et al., 2012). The current study was designed to contribute to that literature by examining how individual differences in executive functioning and vocabulary knowledge contribute to variance in L2 listening performance. Executive functioning represents the range of functions performed by the central executive (the processing component) of the working memory system that are responsible for revising information held in temporary storage as needed for task accomplishment, switching attentional focus among mental representations generated from information processing, and suppressing distractions from influencing task performance (Miyake et al., 2000). It is domain-general, meaning that it is involved in the performance of a wide range of tasks, including language comprehension. Research has shown that individual differences in executive functioning affect L1 performance (Cantin et al., 2016), though it has yet to receive much attention in the L2 literature. To address this scarcity in research, the present study examined executive functioning in the context of L2 listening comprehension.

### L2 Listening Comprehension

L2 listening comprehension is operationalized similarly to Buck’s (2001) definition of the construct. He explains that L2 listening involves being able to “process extended samples of realistic L2 speech, automatically and in real time, to understand linguistic information that is included within a text, and to make inferences based on information that are implicated by the content of the passage” (p. 114). Listening tests assessing comprehension that are operationalized this way measure the ability to identify information explicitly stated within listening texts, and comprehend information implicitly provided in speech (Wagner, 2004). These instruments focus on evaluating higher-level listening skills, so it is important to understand the process listeners go through to arrive at their interpretations of L2 speech.

Imhof (2010) conceives listening comprehension as a recursive structure-building process that places working memory at the center of the sequence. Listeners first select information by filtering out recognizable sounds from irrelevant noise. These sounds are then grouped into meaningful units. Linguistic knowledge plays an important role in these early stages of processing when the mental lexicon is accessed to identify and attach meaning to words which are subsequently organized into a text model of the utterance. The text model represents the information provided within a text and serves as the basis for developing a situation model of the speech (Kintsch, 1998). The situation model represents what the speech is about and is based on inferences drawn from the text model. These inferences provide additional information inherent in the speech, but are not explicitly stated in it. The later stages of processing are happening in working memory, where mental representations of the speech are generated and revised based on their relevance for goal accomplishment by means of an executive function called updating. Imhof (2010) notes that the challenge for listeners is to store representations long enough to be accessed for further processing, while continually updating them when incoming utterances are processed. Further complicating the matter is the potential for interference from inappropriately activated schemas in building structures of the speech. Accurate structures are built when listeners are able to efficiently switch among schemas that are relevant to the input while inhibiting irrelevant schemas. The presence of irrelevant schemas slows the switching function and harms the quality of the situation model being developed.

Throughout the processing sequence, executive functioning plays a central role because it controls what information is selected for attention, aids in the organization of the information by switching among activated representations to generate a text model, and finally facilitates the information-integration process by updating incoming information for goal relevance.

Despite its theoretical significance, executive functioning has been labeled as a peripheral factor as it relates to language ability. Describing how individual listener factors influence language performance, Hulstijn (2015) proposes a core-peripheral model stating that linguistic knowledge, comprised of vocabulary, grammar, and phonological knowledge and the speed at which this knowledge is accessed, explains most variance in language performance for language users at all levels of proficiency. All other factors, including general cognitive abilities, like executive functioning, are peripheral and not as important as linguistic knowledge for language performance. However, the peripheral factors purportedly can contribute more to listening performance for high-proficiency learners than low-proficiency learners. This theory aligns with Cummins’s (1979) threshold hypothesis, which states that language performance is mainly influenced by linguistic knowledge, but that non-linguistic factors become influential as proficiency increases. The limited literature that has examined executive functioning in comprehension appears to support this proposed relationship, though a direct observation has yet to be reported. The current study addressed this gap to examine the relationship among listening comprehension, vocabulary knowledge, and executive functioning.

### Executive Functioning

Executive functioning is operationalized the same as Miyake and Friedman (2012), that is, as updating and shifting. Updating refers to processing representations of input, maintaining them, and revising them as needed for task completion. For L2 listening, new representations are created when an utterance of L2 speech is processed through the language comprehension process. As subsequent utterances are processed, new representations either combine with existing representations being maintained or replace representations based on their relevance to the current task (Morris and Jones, 1990). Shifting refers to switching attentional focus from one schematic representation to another, while inhibiting interference from influencing task performance. This interference includes representations that may have been previously activated from long-term memory to complete an earlier task. Earlier conceptions of executive functioning separate shifting from inhibition (Miyake et al., 2000), but because efficient shifting involves being able to suppress irrelevant representations while switching to those needed for a new task, they are represented as one construct. For L2 listening, completing comprehension tasks requires listeners to switch among the representations generated from language processing as needed to accomplish listening goals (e.g., listening for specific information, listening for gist).

It is unclear how strong the relationship is between updating and comprehension because research has reported mixed results when examining the relationship. This inconsistency in findings may be attributed to differences in linguistic proficiency. Supporting the threshold hypothesis and core-peripheral model, it has been shown that updating is more strongly related to listening performance when listeners have more linguistic resources. For example, Andringa et al. (2012) reported that updating for L1 users was associated with linguistic knowledge (inclusive of vocabulary knowledge, grammatical processing, and segmentation processing), and that both updating and linguistic knowledge explained variance in listening comprehension. In contrast, updating for intermediate level language learners did not correlate with linguistic knowledge and had a weaker relationship with L2 listening comprehension. These findings indicate that listeners with greater linguistic resources are more efficient in updating information and comprehending what they hear than those with less knowledge. In other words, updating explains some variance in listening comprehension when listeners are more proficient language users. Another explanation for the mixed findings may be that the reliability estimates for working memory measures have rarely been reported in these studies (e.g., Brunfaut and Révész, 2014; Vandergrift and Baker, 2015, 2018; Wolfgramm et al., 2016). Because it is unclear if the measures were internally consistent or not, it is possible that the items on the working memory tests may not have consistently measured the same construct, which calls into question the validity of the results.

Similarly, the literature examining the shifting-L2 comprehension relationship has suggested that language users with greater linguistic resources tend to be more skilled at switching (Costa and Santesteban, 2004; Kroll et al., 2008; Bialystok, 2015). Having more knowledge of the target language leads to higher quality representations generated from the input as a result of the language processing cycle. Because the quality of the representations is better, being able to switch among them takes less effort and there are fewer representations competing for attentional focus. In contrast, listeners with limited linguistic resources may be forced to cope with a larger number of incomplete or irrelevant representations remaining from decoding. Navigating among these representations consumes cognitive resources, thus causing representations generated from the input that do receive attentional focus to decay, and ultimately harm comprehension. Because shifting has yet to be explicitly examined along with updating in the L2 listening context, it is unclear how it may relate to listening performance.

### Auditory Vocabulary Size

In addition to executive functioning, auditory vocabulary size was examined to control for language knowledge that purportedly correlates strongly with language performance. The language knowledge construct is more comprehensive than vocabulary, but the current study focused solely on auditory vocabulary size because it accounts for breadth of vocabulary and phonological knowledge. Not including other factors (e.g., grammatical knowledge and access speed) is acknowledged as a limitation of this study. Auditory vocabulary size is operationalized as the ability to recognize target language vocabulary from speech. In many L2 listening studies, vocabulary is measured with vocabulary size tests that use the written format. However, it is important to examine vocabulary size through the same mode as the outcome variable, which in this study is listening comprehension. Doing so allows for phonological knowledge to be accounted for within the vocabulary construct, as opposed to orthographic knowledge that is inherently measured in written tests. Empirical research has consistently reported that auditory vocabulary size shares a relationship with L2 listening comprehension, and that it explains most variance in listening performance when measured alongside other factors. For example, Vandergrift and Baker (2015) reported that auditory vocabulary size shared the strongest relationship with L2 listening performance when measured with auditory discrimination, working memory, metacognition, and L1 vocabulary size for teenage, beginner-level L2 French learners. A similar pattern of results was reported by Vandergrift and Baker (2018), who showed that auditory vocabulary size was the strongest predictor of L2 listening comprehension when modeled along with the same variables as the 2015 article. In both of these studies, auditory vocabulary size explained the most variance in L2 listening performance for the low-level participants, lending support for the core-peripheral model. The present study aims to further test the validity of the core-peripheral model by examining differences in the relationships among L2 listening comprehension, vocabulary size, and executive functioning and whether the vocabulary size may moderate the relationship between executive functioning and listening performance.

### Characteristics of L2 Listening Measures

Characteristics of the listening measures may also influence the relationship among listening comprehension, executive functioning, and vocabulary. Brunfaut and Révész (2014) explain that when listening tests utilize longer listening tracks, it can be expected that executive resources would be more heavily taxed because listeners would need to store large amounts of information from the extended input. This should manifest itself in a correlation between updating and listening measures, but this has yet to be examined. The listening test used in the current study contained longer tracks (68 s to 2 min), which were expected to exceed the short-term memory capacity of the listeners.

The skills measured on the test may also influence the executive functioning and listening comprehension relationship. Listening tests used in empirical studies have typically mirrored Wagner’s (2004) model of listening assessment, where assessments measure the ability to identify information explicitly stated within a spoken text (inclusive of main ideas and details) and to comprehend information implicit in speech (e.g., Tsuchihira, 2007; Andringa et al., 2012; Brunfaut and Révész, 2014; Vandergrift and Baker, 2015, 2018). Of the two, it is expected that items measuring comprehension of implicit information would tax executive resources more since doing so requires listeners to build a mental model of the speech and hold onto it while making connections to what is already known in existing memory to fill in gaps not provided from the input. This has yet to be investigated since most studies have examined listening comprehension using tests that have combined both skills within the same tasks (e.g., Vandergrift and Baker, 2015, 2018).

## The Present Study

The current study examined the relationships among L2 listening performance, updating, shifting, and auditory vocabulary size. Data used to examine the relationships among these factors were taken from a larger study that investigated whether domain-specific knowledge (vocabulary knowledge and topical knowledge) mediated the relationship between L2 listening performance and domain-general cognitive abilities (metacognitive awareness [awareness of (1) oneself as a listener, (2) of a listening task, and (3) of listening strategies], short-term memory [recall of information from temporary memory], and attentional control [shifting]) (Wallace, in press). Specifically, the current study aimed to answer the following research questions.

1. What are the relative contributions of updating, shifting, and vocabulary size to L2 listening performance?
2. Do the contributions of updating and shifting differ for shorter and longer texts?
3. Do the contributions of updating and shifting differ for tasks requiring identification of information explicitly stated within texts and for tasks requiring comprehension of information implicit in texts?
4. In a L2 environment where vocabulary size may be small, does oral vocabulary size moderate the relationship between executive functioning and L2 listening performance?

Supported by the threshold hypothesis (Cummins, 1979) and the core-peripheral model (Hulstijn, 2015), it was expected that vocabulary size would be the strongest predictor of L2 listening performance. Regarding the task characteristics, because executive functions are expected to be more heavily recruited for longer listening texts than shorter, it was expected that updating and shifting would be predictive of listening comprehension for longer texts. The study also expected updating and shifting to be more predictive of tasks requiring comprehension of implicit information than tasks requiring listeners to identify information explicitly stated within texts. Understanding implicit information is more cognitively demanding because it recruits the executive functions to deal with the processing demands of generating a situation model, whereas identifying information within a text relies more on storage of information. Finally, because the relationship between executive functioning and listening performance may depend on vocabulary size, it was expected that vocabulary knowledge would moderate the relationship between listening comprehension and executive functioning, even for low-proficient listeners in this study.

## Materials And Methods

### Participants

In total, 240 first and second year EFL students (aged 15–16) from a private senior high school in Tokyo were invited to participate in the study. The students were arranged in six in-tact classes of 40 students. Of the students asked to participate, 14 elected to withdraw at some point during the data collection and another 17 were eliminated through the data screening process (incomplete data or outliers), leaving 209 (53% female, 47% male) in total. All participants had undertaken at least 3 years of compulsory English education in junior high school (ages 12–14), where they received 4 h of instruction on average per week (MEXT, 2008). In senior high school, the participants received up to 8 h of English instruction per week. Two hours were devoted to explicit grammar instruction, while the remaining 6 h comprised reading, writing, listening, and speaking under an integrated skills syllabus. Students attending this school are typically within a higher socio-economic status than most senior high school students studying in Tokyo. They were expected to be around the Common European Framework of Reference for Languages (CEFR) A2 level. The results from the TOEFL Junior listening test measuring CEFR A2-B2 levels showing they scored an average of 45% (18 out of 40) indicate that they were on the lower end of that scale.

### Instruments

#### L2 Listening

In line with the operational definition of L2 listening, the listening section of a pilot version of the Test of English as a Foreign Language (TOEFL) Junior Standard Test served as the L2 listening performance measure. This paper-based test was designed to measure the language proficiency of English-language learners ranging from below CEFR level A2 to CEFR B2 (ETS, 2018). Content analysis of the 40-item multiple-choice test by a content area expert and the researcher identified half of the items as measuring the ability to identify information provided explicitly in the text and half measuring the ability to comprehend information implicit in the text. Each item and its associated input were coded for whether the answer could be found directly within the text or not. The rater agreement was above 90% and disagreements were discussed until there was full agreement. The first section of the test (17 items) consisted of short monologs and conversations (8–40 s) between school staff members and students and among students themselves. One item was associated with each listening text. Tracks for the second section (23 items) consisted of longer monologs and conversations (68 s to 2 min), with multiple items (three to five) per listening track. Participants could see the questions and answer choices for each associated listening track throughout the test.

#### Updating

Updating was measured using three widely used tests: the Keep-track test (KTU) (Yntema, 1963), the Letter-memory test (LMU) (Morris and Jones, 1990) and the Figural-Spatial 3-back test (FS3B) (Kirchner, 1958). The format of the KTU and LMU were consistent with how they were used in Miyake et al. (2000) and the FS3B in Schmiedek et al. (2009). The language was changed to Japanese to suit the present study’s participants. The other characteristics of the measures mirror those used in Miyake et al. (2000) and Schmiedek et al. (2009).

The KTU required participants to recall the last word for a particular semantic category. Participants saw a sequence of 15 words presented serially. At the same time, two to four semantic categories (countries, clothes, animals, sports) were listed on the bottom of the screen. After all of the words from the trial were presented, participants wrote the last word for each category from the list on answer sheets. The tests included four practice trials (two trials with seven stimuli words and one semantic category, and two trials with 15 stimuli and two semantic categories) and 12 experimental trials (three trials each at two semantic groups, three semantic groups, and four semantic groups with 15 stimuli each).

The LMU required participants to recall only the last four Japanese characters from a sequence of characters. Japanese katakana characters (e.g., ス、ア、イ、ン、マ、etc.) were presented serially for 2000 ms in the middle of the computer screen, with a 500 ms pause between each character presentation. The final four characters did not form meaningful words or phrases in Japanese. The test included three practice trials (two 5-character sequences and one 7-character sequence) and 12 experimental trials (three trials each at 5, 7, 9, and 11 character lengths).

The FS3B required participants to recall the most recent position of boxes on a grid. Participants were presented with a 4 × 4 grid of white boxes in the middle of the screen. One box on the grid turned black for 500 ms and then turned white again for 1500 ms before another box turned black. Participants assessed whether the position of the box that turned black matched the position of the box that turned black three turns before (or three-back). Participants completed two practice trials (10 box positions needing matching judgment) and three experimental trials (21 boxes requiring judgment each trial).

After the experimental trials were completed, a score representing each test was calculated by summing the total number of correct responses for every possible response on the test.

#### Shifting

Shifting was measured using three well-established tests: Number-letter test (NLT) (Rogers and Monsell, 1995), Plus-minus test (PMT), and Global-local test (GLT) (Miyake et al., 2000). The test was administered on computers to collect response and response-time data. The language was changed to Japanese to suit the present study’s participants and the characteristics of the tests are consistent with Miyake et al. (2000).

The NLT asked participants to indicate whether the number of a number-character pair (e.g., 2キ) was even or odd when presented on the top of the screen, and whether the character was a vowel (ア、イ、ウ、エ、オ) or a consonant (カ、キ、ク、ケ、コ) when presented on the bottom of the screen. The test consisted of six trials: number-only trial with pairs shown only at the top of the screen, character only trial with pairs only on the bottom of the screen, and two switch trials with pairs presented clockwise from top left quadrant of the screen to top right, bottom right, and bottom left.

The PMT required participants to switch between adding “two” to a number and subtracting “two” from a number. When numbers were presented in black on the computer screen, they added, and when it was gray, they subtracted. Participants indicated their response using the keyboard. The test consisted of four trials: add only with 34 black numbers, subtract only with 34 gray numbers, and two switch trials with 17 black and gray numbers presented alternatively. The GLT required participants to switch between features of large and small sized figures. Large (global) geometric figures (circles, cross, triangle, square) were presented on screen with lines composed of the same geometric figures (local). Depending on the color of the figure presented, participants counted the number of lines (1 for circle, 2 for cross, 3 for triangle, 4 for square) that composed either the “global” figure (if it was black) or the “local” figure (if it was blue). The test consisted of four trials: global only with 24 black figures, local only with 24 blue figures, and two switch trials with 12 black and blue figures presented alternatively.

After the experimental trials were completed, a shifting efficiency score was calculated for each test by dividing the total number of correct responses for each trial by the mean reaction time of correct trials (Ellefson et al., 2017). This allowed for speed-accuracy tradeoffs to be taken into account. For the purposes of analysis, the efficiency scores were converted to whole numbers by multiplying 100 to them.

#### Auditory Vocabulary Size

Auditory vocabulary size was measured using two sections of the Listening Vocabulary Levels Test (LVLT) (McLean et al., 2015). The words used on the LVLT came from Nation’s (2012) word lists comprising the most frequently used headwords from the British National Corpus and Corpus of Contemporary American English. Nation compiled word lists based on these corpus databases, reduced word families to headwords, and divided them into levels (1000 words per level) based on frequency of occurrence. Only the first 2,000 word level sections of the test were used because a profile of the listening test texts showed that they contained over 94% of words from this level. It was expected that this level would be needed to have sufficient lexical coverage for the listening test. In terms of format, the test consisted of two sections: one section each for the first two 1000 word levels, with 24 words per section. Each word was spoken once, followed by a sentence that did not reveal the meaning of the word. Participants matched the English word they heard to the corresponding word in Japanese (the L1). After the test, a total score for both sections was calculated.

### Data Collection Procedures

After receiving ethical clearance and permission to conduct the study from the high school administration, students were recruited from their English classes by one of the researchers and a teacher. Students who provided parental consent and agreed to participate in the study completed the instruments after school on four separate days over a 3 week span. Each test was administered in groups of up to 40 students. The listening and vocabulary tests were delivered in their paper-and-pencil format in a classroom and took 40 and 20 min to complete, respectively. For the listening test, following recommendations by Educational Testing Service, the instrument developer, participants heard each audio once and recorded their responses on their corresponding answer sheet. Similarly, as recommended by McLean et al. (2015), participants heard each vocabulary word and corresponding sentence once and indicated their response on their answer sheet. The responses were inputted into SPSS version 24 (IBM, 2016) for subsequent analysis. A research assistant verified the accuracy of the data entry by manually checking the match between test responses and data input into SPSS.

The executive functioning tests were administered in a computer lab. Groups of up to 40 participants completed the three shifting tests on 1 day and the updating tests on a different day. The researcher led a demonstration of each test before directing the participants to complete them. It took 40 min to complete all three updating and all three shifting tests. After completing each test, participants took a 5 min break. All six tests were delivered on computers using E-Prime 2.0 (Schneider et al., 2002). For the shifting tests and FS3B, responses and response times were collected. For the KTU and LMU updating tests, participants indicated their responses on an answer sheet. The responses and their associated times were exported to SPSS for subsequent analysis. A research assistant verified the accuracy of these responses by matching the test responses with the input response in SPSS.

### Data Analysis

Variables from each test were created for analysis. For the listening test, five variables were computed. One variable consisted of the total score on the TOEFL Junior listening test. Two variables divided the listening items by text length. One measured short texts (17 items) and another measured long texts (23 items). Two other variables divided the listening items by skill measured. One measured the ability to comprehend explicitly stated information (20 items) and another measured comprehension of implicit information (20 items). Descriptive statistics and reliability estimates were calculated for each measure to provide evidence of normality and internal consistency. Outliers were identified by examining the inter-quartile range of scores. Z-scores were calculated for the variables and if their values were larger than the absolute value of 2.68, they were considered an outlier and removed from the analysis. The skewness and kurtosis values were inspected after the outliers were removed. Variables with values smaller than 2.0 were considered normally distributed (Field, 2009). Multivariate outliers also were inspected by calculating the Mahalanobis distances for the variables in the study and comparing them to a chi-square distribution with the same degrees of freedom. If the *p*-value of the right tail of the chi-square distribution was below 0.001, then multivariate outliers would be present and subsequently removed. To inspect multivariate normality, Mardia’s coefficient (Mardia, 1970) was calculated. Values outside the absolute value of 3.0 are considered non-normal (Bentler, 2006). To verify the unidimensionality of the variables, they were subjected to Principal Components Analysis of Residuals, the statistical procedure in Rasch Modeling that identifies the difference in the amount of variance that is explained by the Rasch model with variance left unexplained in the model, called Rasch residuals. To determine the difference in variance, Winsteps (Linacre, 2016) produces Eigenvalues and percentage of variance explained by both the Rasch model and Rasch residuals (called Contrasts in Winsteps). Larger Eigenvalues (above 2.0) with large percentages of variance explained by Contrasts would indicate the instrument was multidimensional. However, if the Eigenvalues of the Rasch model are up to three times in excess to that of the Contrast Eigenvalues, the instrument can still be considered unidimensional.

**FIGURE 1:**
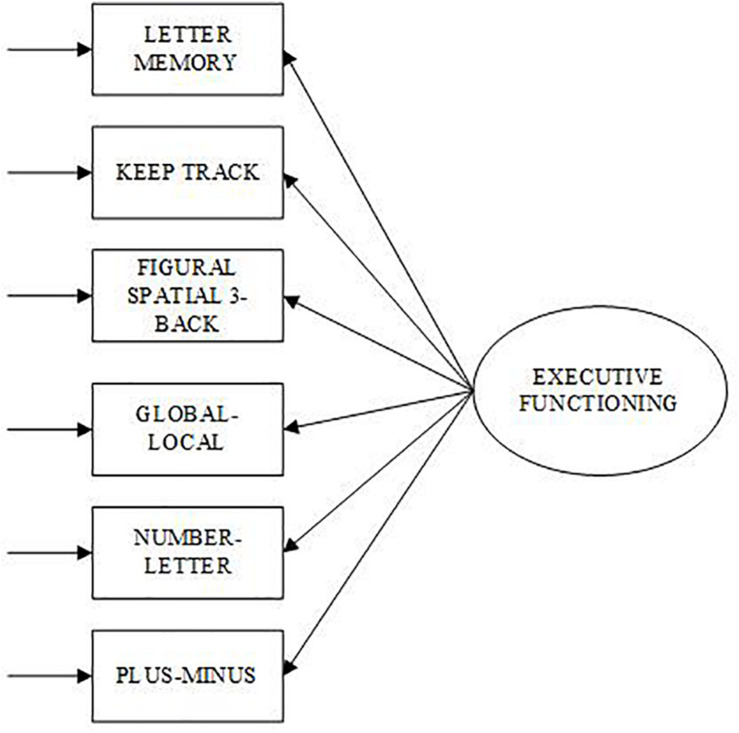
Single factor confirmatory model of Executive Functioning. Ovals represent latent variable and rectangles represent observed variables.

**FIGURE 2:**
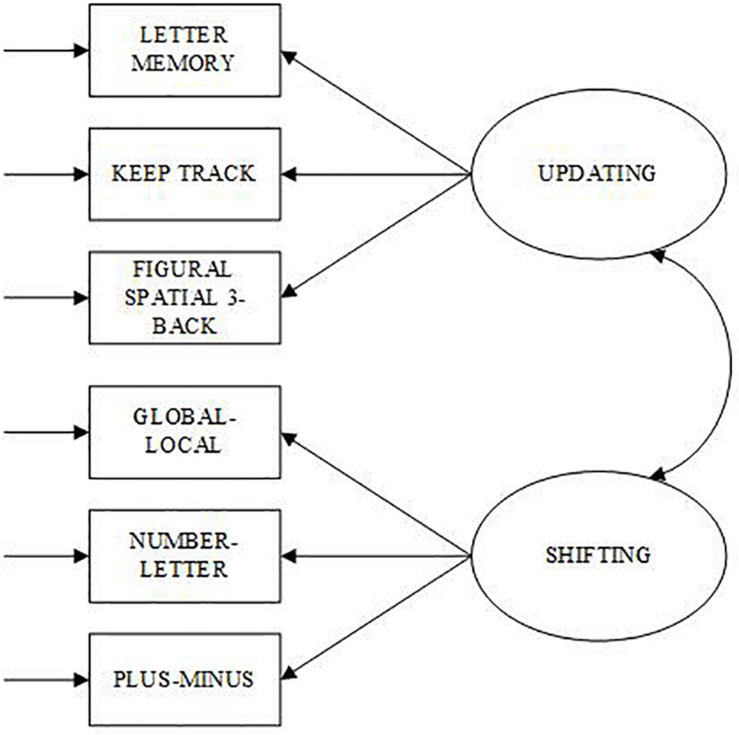
Two-factor confirmatory model of Executive Functioning. Ovals represent latent variables and rectangles represent observed variables.

To test the dimensionality of the updating and shifting factors, and to verify that the executive functions are separate, confirmatory factor analysis (CFA) was conducted using MPlus (version 8.4) (Muthen and Muthen, 1998-2019). Two measurement models were examined: Single factor and Two-factor model. One factor was regressed onto all six executive function variables for the Single factor model (see Figure 1). For the Two-factor model (see Figure 2), an updating factor was regressed onto three updating variables (KTU, LMU, FS3B) and a shifting factor was regressed onto three shifting variables (GLT, NLT, PMT). A correlation parameter was set between updating and shifting factors. The Maximum Likelihood estimation method was used for identification and the factor variances for the latent variables were set to 1.0, allowing the path coefficients to be freed.

To test which model fit the data better, the model fit statistics were compared and a chi-square difference test was run. Kline (2016) suggests that model fit is considered good when the Comparative Fit Index (CFI) is above 0.900, the Root Mean Square Error of Approximation (RMSEA) is below 0.05 and the Standardized Root Mean-Square Residual (SRMR) is below 0.08. The Bayesian Information Criteria (BIC), a statistic that is used to compare models that is sensitive to degrees of freedom, sample size, and model complexity, was also consulted. Lower BIC values indicate more parsimony, and therefore, better fitting model. Vocabulary size was added to the better fitting confirmatory model to confirm the factor structures of the predictors (EF-VS model). In the model, vocabulary size was correlated with updating and shifting factors.

To answer the first research question, L2 listening was regressed onto the updating, shifting, and vocabulary size factors. To answer the second and third questions, the listening factor was divided into two different subsets of the listening construct: one subset for length of text and the other for type of information requiring comprehension on the test. For length, variables for short and long texts were regressed onto the updating, shifting, and vocabulary factors. To answer the third research question examining comprehension of information type, the variables representing comprehension of explicit information (20 items) and implicit information (20 items) items were regressed onto the updating, shifting, and vocabulary size factors. Fit statistics were consulted to evaluate how closely the data fit the models. To answer the final research question, two moderator variables consisting of vocabulary size and updating and vocabulary size and shifting were created. The structural model was re-run twice with the moderator variables included, respectively. If the moderator variable explained variance in listening performance, then an interaction would be present.

## Results

The descriptive statistics, reliability estimates, and Principal Components Analysis of Residuals estimates are presented in Table 1. The skewness and kurtosis values of the variables show that they all were within the absolute value of 2.0 and the Mardia coefficient was within the absolute value of 3.0, indicating the data was approximately normal. Coefficient alpha for each of the measures indicates an acceptable level of internal consistency for the variables. Principal Components Analysis of Residuals indicated that the variables were unidimensional. Though the vocabulary, LMU, KTU, NLT, and PMT variables had Eigenvalues above 2.0, the percentage of variance explained by the Rasch model was over three times that explained by the first contrast.

**TABLE 1:** Descriptive statistics, reliability estimates, and Principal Components Analysis of Residuals of Rasch dimension and Unexplained Variance for all variables (*n* = 209).

Measure	Mean	*SD*	Max value	Skewness	Kurtosis	Reliability	Rasch dimension (EV)	Unexplained variance: first contrast (EV)
L2 listening	18.07	6.77	40	0.552	–0.438	0.823	20.6%(10.40)	4.5% (2.28)
Explicit	9.01	3.77	20	0.392	–0.452	0.705	20.5%(5.17)	7.5% (1.88)
Implicit	9.05	3.58	20	0.317	–0.338	0.690	25%(6.67)	6.2% (1.66)
Short	8.80	3.49	17	0.174	–0.655	0.718	24.5%(5.39)	7.2% (1.60)
Long	9.28	4.01	23	0.498	–0.329	0.711	20.5%(5.92)	6.2% (1.79)
KTU	18.88	3.62	27	–0.587	0.830	0.643	21.8%(7.54)	5.4% (1.87)
LMU	38.91	6.36	48	–0.708	0.292	0.845	22.3%(13.77)	5.5% (3.40)
FS3B	42.18	12.38	72	–0.931	0.670	0.925	18.7%(14.53)	3.4% (2.66)
NLT	3.65	0.817	12	0.384	0.476	0.757	47.6%(32.67)	3.7% (2.56)
PMT	1.35	0.299	7	0.525	0.787	0.836	30.2%(14.68)	4.9% (2.37)
GLT	3.06	0.629	16	0.321	0.149	0.774	44.0%(18.88)	4.5% (1.94)
VS	38.99	3.81	48	–0.489	0.626	0.640	34%(21.02)	3.8% (2.99)

*Explicit, explicit information items; Implicit, implicit information items; Short, items associated with short texts; Long, items associated with long texts; KTU, keep-track test; LMU, letter-memory test; FS3B, figural-spatial 3-back test; NLT, number-letter test; PMT, plus-minus test; GLT, global-local test; VS, vocabulary size; Max score, maximum possible score; EV, eigenvalues.*

Intercorrelations among the variables show that not all of them were correlated with one another (Table 2). The listening and vocabulary variables were associated with each other, but the strength of the correlations was weaker than anticipated. None of the updating variables correlated with the listening variables, and only two shifting variables (NLT and GLT) correlated with listening variables.

**TABLE 2:** Correlation matrix for the variables (*n* = 209).

Variable	1	2	3	4	5	6	7	8	9	10	11
L2 listening	1										
Explicit	0.926**	1									
Implicit	0.918**	0.700**	1								
Short	0.888**	0.763**	0.878**	1							
Long	0.917**	0.901**	0.787**	0.631**	1						
KTU	0.085	0.048	0.111	0.102	0.057	1					
LMU	0.130	0.126	0.112	0.118	0.114	0.290**	1				
FS3B	0.118	0.115	0.102	0.113	0.104	0.183**	0.082	1			
NLT	0.159*	0.163*	0.129	0.151*	0.135	0.180**	0.105	0.051	1		
PMT	0.107	0.086	0.111	0.142*	0.057	0.095	0.057	0.148*	0.249**	1	
GLT	0.154*	0.137*	0.147*	0.137*	0.142*	0.163*	0.073	0.107	0.450**	0.311**	1
VS	0.439**	0.388**	0.423**	0.437**	0.362**	0.147*	0.159*	0.116	0.094	0.097	0.083

* p < 0.05; ** p < 0.01; Explicit, explicit information items; Implicit, implicit information items; Short, items associated with short texts; Long, items associated with long texts; KTU, keep-track test; LMU, letter-memory test; FS3B, figural-spatial 3-back test; NLT, number-letter test; PMT, plus-minus test; GLT, global-local test; VS, vocabulary size.

### Confirmatory Factor Analysis

Results presented in Table 3 indicated that the Two-factor model fit the data better than the Single factor model. This was confirmed by the chi-square difference test showing that the Two-factor model was statistically different from the Single factor model (Δ*x*^2^ = 18.78, Δdf = 1, *p* < 0.01), and therefore fits the data better. The results also showed that the two executive functions shared a moderate relationship (*r* = 0.368, *p* < 0.01). In line with Lee et al. (2013), these results support the expectations that the two executive functions were separable for the mid-adolescent participants in this study. They also support Miyake and Friedman’s (2012) contention that updating and shifting are unified (in that they shared a relationship) yet diverse (the relationship was not strong). Vocabulary size was then added to the Two-factor model and the results show good fit to the EF-VS model of the predictors.

**TABLE 3:** Fit indices for Single factor, Two-factor, and EF-VS measurement models.

Model	*x*^2^	*df*	*p*-value	*CFI*	*RMSEA*	*BIC*	*SRMR*
Single factor	22.945	9	0.006	0.856	0.086	5089.427	0.058
Two-factor	4.173	8	0.841	1.000	0.000	5075.998	0.025
EF-VS	6.064	12	0.913	1.000	0.000	6239.489	0.025

*x^2^, chi squared statistic; df, degrees of freedom; CFI, comparative fit index; RMSEA, root mean square error of approximation; BIC, Bayesian Information Criteria; SRMR, standardized root mean-square residual.*

### Structural Equation Modeling

**FIGURE 3:**
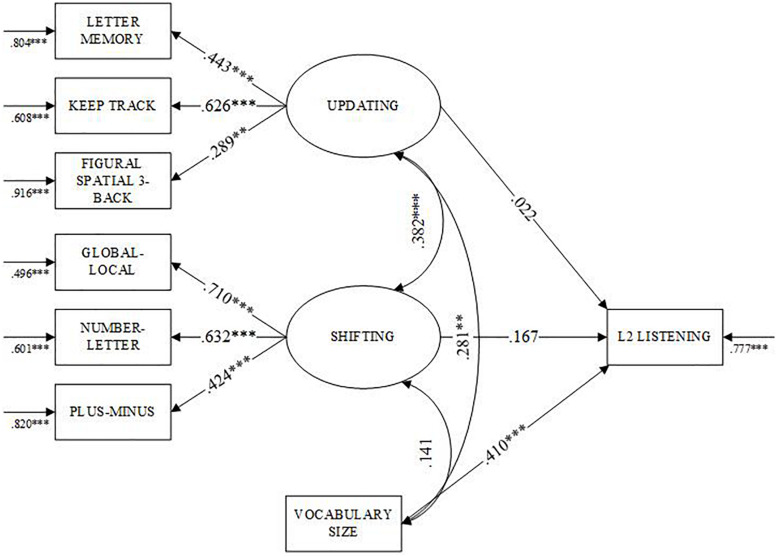
Standardized parameters of the SEM model of L2 listening comprehension. **p < 0.01, ***p < 0.001. Ovals represent latent variables and rectangles represent observed variables.

**FIGURE 4:**
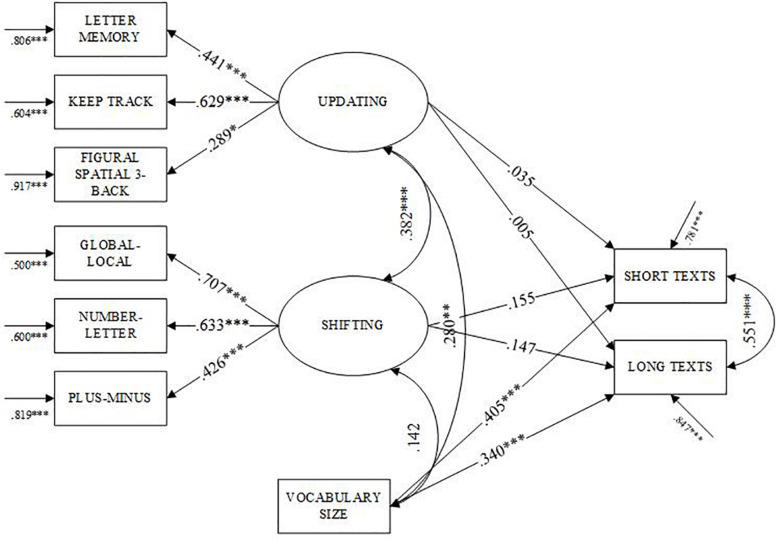
Standardized parameters of the SEM model of short and long texts. *p < 0.05, **p < 0.01, ***p < 0.001. Ovals represent latent variables and rectangles represent observed variables.

**FIGURE 5:**
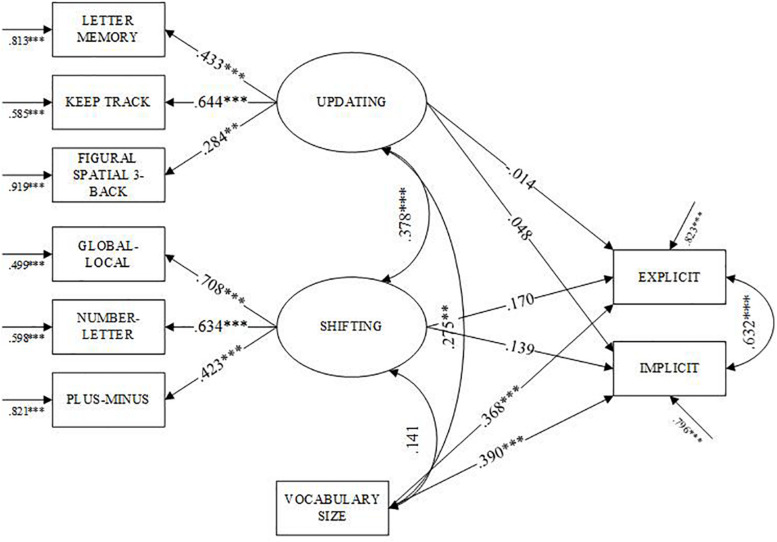
Standardized parameters of the SEM model of explicit and implicit comprehension. **p < 0.01, ***p < 0.001. Ovals represent latent variables and rectangles represent observed variables.

Table 4 presents results from the SEM analyses. The results for the first research question show that the data fit the L2L model well (see Figure 3): non-significant *x*^2^(16) = 7.449, *CFI* = 1.00, *RMSEA* = 0.00, *BIC* = 7607.667, and *SRMR* = 0.026. Of the three variables, vocabulary size was the only one that was predictive of L2 listening performance (β = 0.410, *p* < 0.01). Vocabulary also correlated with updating (*r* = 0.281, *p* < 0.01), but not shifting. The SEM results for research question two show good fit to the Short-Long model (see Figure 4): *x*^2^(20) = 9.792, *CFI* = 1.00, *RMSEA* = 0.00, *BIC* = 8420.937, and *SRMR* = 0.026. Vocabulary was a stronger predictor of shorter texts (β = 0.405, *p* < 0.001) than longer texts (β = 0.340, *p* < 0.001). The SEM results for the third research question also show good fit to the Explicit-Implicit model (see Figure 5): *x*^2^(20) = 10.045, *CFI* = 1.00, *RMSEA* = 0.00, *BIC* = 8377.739, and *SRMR* = 0.027. Vocabulary was the only predictor of the listening variables, explaining more variance in scores for implicit information items (β = 0.390, *p* < 0.01) than explicit information items (β = 0.368, *p* < 0.01). In every model, neither updating nor shifting were predictive of L2 listening comprehension after controlling for vocabulary size. However, updating and shifting shared a moderate relationship with one another. Regarding the final research question, the results showed that the vocabulary size did not moderate the relationship between listening performance and updating (β = 0.112, *p* = 0.240) or shifting (β = 0.018, *p* = 0.805).

**TABLE 4:** Fit indices for structural models.

Model	*x*^2^	*df*	*p*-value	*CFI*	*RMSEA*	*BIC*	*SRMR*
L2 listening	7.449	16	0.964	1.000	0.000	7607.667	0.026
Short-long	9.792	20	0.972	1.000	0.000	8420.937	0.026
Explicit-implicit	10.045	20	0.967	1.000	0.000	8377.739	0.027

*L2L, L2 listening as latent variable; Explicit-Implicit, L2 listening as explicit and implicit item observed variables; Short-long, L2 listening as short and long text observed variables; x^2^, chi squared statistic; df, degrees of freedom; CFI, comparative fit index; RMSEA, root mean square error of approximation; BIC, Bayesian Information Criteria; SRMR, standardized root mean-square residual.*

## Discussion

### Listener Characteristics

The first research question aimed to examine the relationship among L2 listening performance, updating, shifting, and vocabulary size. The results showed that only vocabulary size was associated with better listening performance, and that neither updating nor shifting were. These results support the core-peripheral model of language proficiency, which states that language knowledge is most important for language performance, and peripheral factors, like executive functioning, are less important (Hulstijn, 2015). The findings align with earlier studies showing that individual differences in working memory, of which executive functioning largely comprises, fails to predict L2 listening comprehension, but that linguistic knowledge in general (Andringa et al., 2012), and vocabulary size in particular (Vandergrift and Baker, 2015, 2018; Wolfgramm et al., 2016) does. For example, Vandergrift and Baker (2015) reported that vocabulary size was predictive of L2 listening performance, but working memory was not for teenage French immersion students with a limited vocabulary size (38% on a vocabulary size test). Explaining similar results for younger participants, Vandergrift and Baker (2018) speculated that the low vocabulary prevented executive resources from aiding in comprehension. They characterized the relationship between working memory and vocabulary as being developmentally linked, stating that efficiency in using executive resources improves alongside increases in language proficiency and as these two increase, comprehension improves. A similar explanation may be offered for the current study, since the young participants were of limited vocabulary size. van Zeeland and Schmitt (2012) set criteria for good comprehension of L2 spoken texts at knowledge of around 90–95% of the vocabulary. However, the mean scores of the vocabulary test (Table 1) show that the participants knew only about 81% of words at the two-thousand vocabulary level, which is well below the threshold required for good comprehension of the listening test texts containing 98% of words from that level. Because the participants were below the threshold of knowledge needed for adequate comprehension, most of their cognitive resources were likely spent in early stages of language processing working out what words they heard. This would limit how useful the executive resources would be in later stages of processing when mental representations were switched among and revised to generate a mental model of the speech. If the listeners were unable to accurately or completely decode words, the executive processes would not be very useful for comprehension because switching among and updating low quality and quantity representations would not generate an accurate or complete mental model of speech.

Another explanation is that the participants’ executive functions were not sufficiently developed to be of use during the listening tasks. It has been claimed that executive functioning may not come to maturity until adulthood (Rose et al., 2011), so it is possible that for the young teenage participants in this study, their updating and shifting abilities may have been undeveloped. The descriptive statistics show that this may have been the case for shifting, since the participants were not very skilled in shifting their attentional focus from one task to another. They were only 30% efficient at switching between numbers and characters, 19% efficient at switching between adding and subtracting numbers, and 19% efficient at switching between shape sizes. These results can be interpreted to mean that when the participants accurately completed tasks, they were slow in switching their attentional focus from one task to another and that when they quickly switched to new tasks, they were less accurate in completing them. This slow and effortful shifting would have posed challenges for listeners because they did not control the pace of speech or duration of the tasks. The TOEFL Junior had 23 items that were associated with six audio tracks (three to four items per track). Participants had to listen to the audio and shift their attentional focus between reading and answering the multiple-choice items and listening for information. Being slow in answering questions about information early in the audio track (first two out of three/four items) may have helped them accurately answer those questions, but they likely would have missed important information given later in the audio that was needed to answer other questions. Results from a paired-samples *t*-test conducted on the TOEFL Junior test items supports this claim and showed that for the multiple-item tasks, the listeners more accurately answered the first two items within a single track (12 items: *M* = 5.49, *SD* = 2.56) than the last items (11 items: *M* = 3.79, *SD* = 2.04), *t*(208) = 10.678, *p* < 0.01. Had shifting resources been more efficient, it is possible that the participants’ listening performance would have been better. However, it appears that their shifting resources were too limited to be of much help for these listeners.

Updating also failed to share a relationship with listening comprehension, but it appears to have been sufficiently developed. The listeners performed moderately well on the updating tests, recalling 70% of the word stimuli, 81% of the character stimuli, and 67% of the figural-spatial stimuli. This means that they were somewhat accurate in being able to revise varied types of information in their short-term memory. It is therefore curious as to why updating was not important for listening performance, especially when doing so was expected to play a key role in the listening comprehension process (Imhof, 2010). The nature of the representations that are being updated may be a reason. In order for updating to aid in comprehension, listeners may need to be efficient in updating representations of the target language. When the representations are different from the target language, like numbers, first language characters, and figures that were measured by the updating tasks, they may not be as helpful for lower-level listeners in comprehending speech. Because the current study did not measure the ability to update target language speech, this was not observed and can be treated as a limitation of the study.

### Listening Test Lengths and Skills

The second research question investigated if the contributions of updating and shifting would differ for longer or shorter text lengths. It was expected that longer listening tracks would engage the executive functions more than shorter tracks since listeners would need to revise more information from the input and switch among more mental representations to generate a mental model of the text. However, similar to the results for L2 listening performance overall, only vocabulary size explained variance in listening comprehension for both lengths (long texts: β = 0.340, *p* < 001; short texts: β = 0.405, *p* < 0.001). These results suggest that when vocabulary is controlled for, executive functions do not influence listening performance, regardless of text length. It has been reported that working memory (memory and executive functions) failed to explain variance in listening comprehension for short texts when controlling for vocabulary size (Vandergrift and Baker, 2018) and linguistic knowledge (grammar and vocabulary) (Andringa et al., 2012). The results of the present study indicate that a similar pattern of relationships may exist for the executive functions of working memory as well, since the updating and shifting executive functions failed to explain variance in comprehension beyond what was explained by vocabulary size.

It may be that the shorter texts did not extend beyond the participants’ memory capacity, meaning that they could remember all of the information without having to revise what they held in their memory and limiting how many mental representations were needed to be switched among. Nearly half of the items of the TOEFL Junior Standard test (17 of the 40 items) utilized short listening tracks that were around 12–40 s long and involved little discourse beyond three to four sentences. For these texts, it is possible that the participants remembered everything that was said. However, this may not explain the results for the longer texts. The other half (23 of the 40 items) of the items were associated with texts ranging from 68 s to 2 min and it would be challenging to remember all of the information provided in these longer pieces of discourse. The executive functions may not have been engaged during these longer texts because having the answer sheet with the questions and answer options available throughout the test reduced the cognitive load required for listening. Participants could have written key points from the texts down on their answer sheets as they listened and/or marked key terms in the question and answer choices as they followed along with the audio. This would have eliminated the need to hold all of the information provided in the texts in their temporary memory and essentially exported the information from the memory system to the paper. Future studies may consider addressing this by examining if the executive functions share a relationship with listening performance when the answer choices are provided and when they are not.

The third research question examined if the contribution of updating and shifting would differ depending on the skills measured on the listening test. It was expected that requiring listeners to comprehend implicit information provided within a text would tax the executive functions. However, updating and shifting did not explain variance in the explicit or implicit listening item scores beyond that explained by vocabulary size. These findings are consistent with Vandergrift and Baker (2015, 2018) who also measured the ability to understand explicit and implicit information in L2 speech for teenage language learners. The multiple-choice response format on the listening tests in these earlier studies and the present one may have contributed to the consistent findings. It has been suggested in the literature that multiple-choice response format may overload cognitive resources and limit comprehension because it introduces the construct-irrelevant factor of reading comprehension by requiring test takers to read and comprehend the questions and answer choices in addition to holding information in memory as they listen (Brunfaut and Révész, 2014). However, the opposite is proposed here as an explanation for the executive functions not sharing a relationship with listening performance. The premise of providing listeners with the goals of a listening task beforehand in order to signal what they should focus on as they listen is consistent with real-world listening events, where implicit or explicit listening goals drive what is attended to in speech. On assessments, this is taken in the form of providing the questions and answer choices before the listening begins. However, when these are provided, it alerts listeners to the specific language they should be listening for in addition to the goal of listening. In this way, the key words given in the questions and answer choices likely activate their prior knowledge before the listening track begins, thus reducing the amount of new representations the listeners needed to generate from the input and overall cognitive load. Executive functioning would therefore be of limited use because the relevant representations needed for comprehension have been pre-activated before the listening started. Listeners would simply narrow their focus on key terms as they listened and link what they heard to what was already activated.

### Moderation

The final research question examined if the influence of executive functioning on listening performance is dependent upon language knowledge for learners of low proficiency levels. The results showed that vocabulary size did not moderate the relationship between listening comprehension and either updating or shifting. It is likely that the vocabulary size was too low for the moderation effect to be detected. For the data to exhibit interaction, participants who are well above the threshold of vocabulary size needed for comprehension of the texts would need to be included in the sample. The participants were well below that threshold. In order to detect this possible interaction, future studies are encouraged to include participants with a larger vocabulary size. Another explanation for this may be that auditory vocabulary size alone did not moderate the relationship. Though Mecartty (2000) reported that vocabulary explained much more unique variance in L2 listening comprehension than grammar, it is possible that not including grammar or other aspects of linguistic knowledge (e.g., vocabulary depth, grammar, speed of accessing language knowledge) may have limited the extent to which language knowledge influenced the executive functioning and listening comprehension relationship. The results may therefore be interpreted to mean that auditory vocabulary knowledge failed to moderate the relationship between executive functions and listening performance. This should be understood as a limitation of the study and future research is encouraged to include grammar, depth of vocabulary knowledge, and access speed when examining whether language knowledge moderates the effects of peripheral factors for listening comprehension.

## Conclusion

To conclude, for the Japanese EFL participants in this study, having a larger auditory vocabulary size was most important for comprehending the L2 speech. Features of the listening test, namely the text lengths and skills measured, did not affect the contributions that updating and shifting made to L2 listening performance. These findings may be attributed to the limited linguistic resources of the participants, as the input may have been beyond the listeners’ threshold of linguistic knowledge and thereby preventing the executive functions from having much influence on comprehension. If there is insufficient existing knowledge to resolve problems presented by the incoming information, no amount of executive function is going to help. The results also showed that vocabulary size did not moderate the relationship between listening comprehension and executive functioning. Altogether, the findings provide partial support for the core-peripheral model of language proficiency, showing that vocabulary size was most important for listening performance, but that the executive functions may not explain variance in comprehension regardless of how many words are known.

This study is not without its limitations. First, the limited sample size and narrow scope in which the data was collected limit the interpretations of the study. The data was collected from a single location with a homogenous group of participants in Japan. In order to generalize the findings to the broader EFL population, the study would need to be replicated in varied contexts. Also, future studies may consider examining the relationships among the executive functions and listening comprehension for participants with a wider range of proficiency levels. This study looked narrowly at lower-level learners and concluded that the limited linguistic resources prevented the executive functions from sharing variance in listening performance. To provide a more comprehensive view of the relationships among the variables examined in this study, and to further test the core-peripheral language proficiency model, future studies may recruit participants from higher and moderate level proficiencies. Future research may also consider utilizing a listening measure that incorporates a multiple-choice format that does not provide the answer choices before the listening starts. To avoid a possible priming effect, where the vocabulary needed for the listening is activated before the listening track plays, it is recommended that listening tests provide only the questions prior to listening and reveal the answer options after the listening track has completed. This will give the listeners a goal to listen for, but minimize their lexical activation. This kind of task may be considered more authentic in that it would require listeners to generate new representations of the input as they listen, similar to a realistic listening encounter. Overall, the findings from this study contribute empirical evidence for the relationship between L2 listening comprehension and executive functions, a novel conceptualization of the working memory construct.

## Data Availability Statement

The datasets generated for this study are available on request to the corresponding author.

## Ethics Statement

The studies involving human participants were reviewed and approved by Nanyang Technological University. Written informed consent to participate in this study was provided by the participants’ legal guardian/next of kin.

## Author Contributions

MW and KL conceived the study. MW carried out the experiment, including instrument design, data collection, analysis, and interpretation and took the lead in writing the manuscript. KL provided critical feedback and helped shape the research, including its design, analysis, results interpretation, and manuscript development.

## Conflict of Interest

The authors declare that the research was conducted in the absence of any commercial or financial relationships that could be construed as a potential conflict of interest.
